# A Framework for Local Mechanical Characterization of Atherosclerotic Plaques: Combination of Ultrasound Displacement Imaging and Inverse Finite Element Analysis

**DOI:** 10.1007/s10439-015-1410-8

**Published:** 2015-09-23

**Authors:** Ali C. Akyildiz, Hendrik H. G. Hansen, Harm A. Nieuwstadt, Lambert Speelman, Chris L. De Korte, Antonius F. W. van der Steen, Frank J. H. Gijsen

**Affiliations:** Biomechanics Lab, Department of Biomedical Engineering, Thoraxcenter, Erasmus Medical Center, PO Box 2040, 3000 CA Rotterdam, The Netherlands; Medical UltraSound Imaging Center (MUSIC), Department of Radiology and Nuclear Medicine, Radboud University Medical Center, Nijmegen, The Netherlands; Department of Applied Sciences, Delft University of Technology, Delft, The Netherlands; Department of Mechanical, Aerospace and Nuclear Engineering, Rensselaer Polytechnic Institute, Troy, USA

**Keywords:** Material properties, Atherosclerotic plaque, Inverse method, Ultrasound

## Abstract

Biomechanical models have the potential to predict plaque rupture. For reliable models, correct material properties of plaque components are a prerequisite. This study presents a new technique, where high resolution ultrasound displacement imaging and inverse finite element (FE) modeling is combined, to estimate material properties of plaque components. Iliac arteries with plaques were excised from 6 atherosclerotic pigs and subjected to an inflation test with pressures ranging from 10 to 120 mmHg. The arteries were imaged with high frequency 40 MHz ultrasound. Deformation maps of the plaques were reconstructed by cross correlation of the ultrasound radiofrequency data. Subsequently, the arteries were perfusion fixed for histology and structural components were identified. The histological data were registered to the ultrasound data to construct FE model of the plaques. Material properties of the arterial wall and the intima of the atherosclerotic plaques were estimated using a grid search method. The computed displacement fields showed good agreement with the measured displacement fields, implying that the FE models were able to capture local inhomogeneities within the plaque. On average, nonlinear stiffening of both the wall and the intima was observed, and the wall of the atheroslcerotic porcine iliac arteries was markedly stiffer than the intima (877 ± 459 vs. 100 ± 68 kPa at 100 mmHg). The large spread in the data further illustrates the wide variation of the material properties. We demonstrated the feasibility of a mixed experimental–numerical framework to determine the material properties of arterial wall and intima of atherosclerotic plaques from intact arteries, and concluded that, due to the observed variation, plaque specific properties are required for accurate stress simulations.

## Introduction

Atherosclerotic plaques are characterized by local thickening of the arterial vessel wall, mainly caused by lipid and inflammatory cell infiltration, smooth muscle cell migration and proliferation and extracellular matrix buildup.^11^ Some atherosclerotic plaques have a higher chance of causing clinical events and are therefore called vulnerable plaques. Vulnerable plaques are morphologically characterized by a large lipid core and a relatively thin fibrous cap, separating the lipid core from the lumen.^39^ Rupture of the cap exposes the content of the lipid core to the blood stream and leads to intraluminal thrombosis. Thrombosis triggered by plaque rupture is the predominant cause of myocardial infarction and stroke.^44^

Today means of accurately predicting the rupture risk of a plaque are still lacking. Biomechanical studies showed that high stress regions in atherosclerotic plaques correspond to rupture locations.^9^,^37^ The rationale behind this is that rupture occurs when the stress at a certain location inside the cap exceeds the local cap strength. Biomechanical models that are used to compare computed cap stresses with cap strength have the potential to improve plaque rupture risk assessment.^18^ For accurate stress calculations, biomechanical plaque models not only rely strongly on the plaque geometry, but also on the material properties of plaque components.^2^,^35^ However, experimental data for plaque properties are scarce and available data span a wide range.^1^,^45^

One of the underlying reasons for the wide spread in the experimental data is that commonly applied testing methods generally involve loss of plaque integrity. By testing atherosclerotic plaques with *in vivo* deformation measurements or *ex vivo* inflation tests on the other hand, the structural integrity of the tissue is not compromised. *Ex vivo* inflation tests have been widely used to estimate material properties of healthy vessels.^19^,^43^ Generally, the deformation of the outer border of the vessel wall was measured. The vessel was modeled as a thin walled cylinder and the material properties were obtained by solving the problem analytically.^14^,^22^,^36^ Due to the complex morphology of atherosclerotic plaques, determination of the material properties cannot be done with analytical solutions and requires more advanced approaches such as inverse FE analysis.

With inverse FE analysis, material properties can be estimated by adjusting material parameters in the FE simulations iteratively and minimizing the difference between the computed and measured deformations. The feasibility of this methodology was demonstrated using synthetic, simulated inflation test results as an input.^5^,^12^,^20^,^21^,^26^ Other groups used the inverse FE based on global plaque deformation measurements, using either intravascular ultrasound^8^,^15^,^32^ or magnetic resonance imaging.^28^ The main drawback of using global deformation measurements is that the underlying FE model cannot incorporate multiple plaque components. Baldewsing *et al.*^3^ applied intravascular ultrasound to obtain local deformation measurements, and they were able to demonstrate the feasibility of the method to generate elasticity maps of coronary plaques. The study focused on *in vivo* applications to determine the material properties for small incremental strain values, and the shape that individual plaque components could adopt to was restricted. Finally, Beatty *et al.* subjected aortic segments to inflation tests to determine material properties using optical techniques to measure the local displacement,^4^ thus limiting the future utilization of this technique in a clinic setting.

In this study the feasibility of a novel hybrid framework for mechanical characterization of atherosclerotic plaques based on *ex vivo* inflation tests is demonstrated. The method combines high resolution ultrasound deformation measurements and plaque morphology information with inverse FE analysis to compute local, nonlinear material properties of arterial wall and intima of atherosclerotic plaques. The feasibility of this framework was demonstrated by characterizing the properties of advanced atherosclerotic plaques from porcine iliac arteries.

## Methods

**Figure 1:**
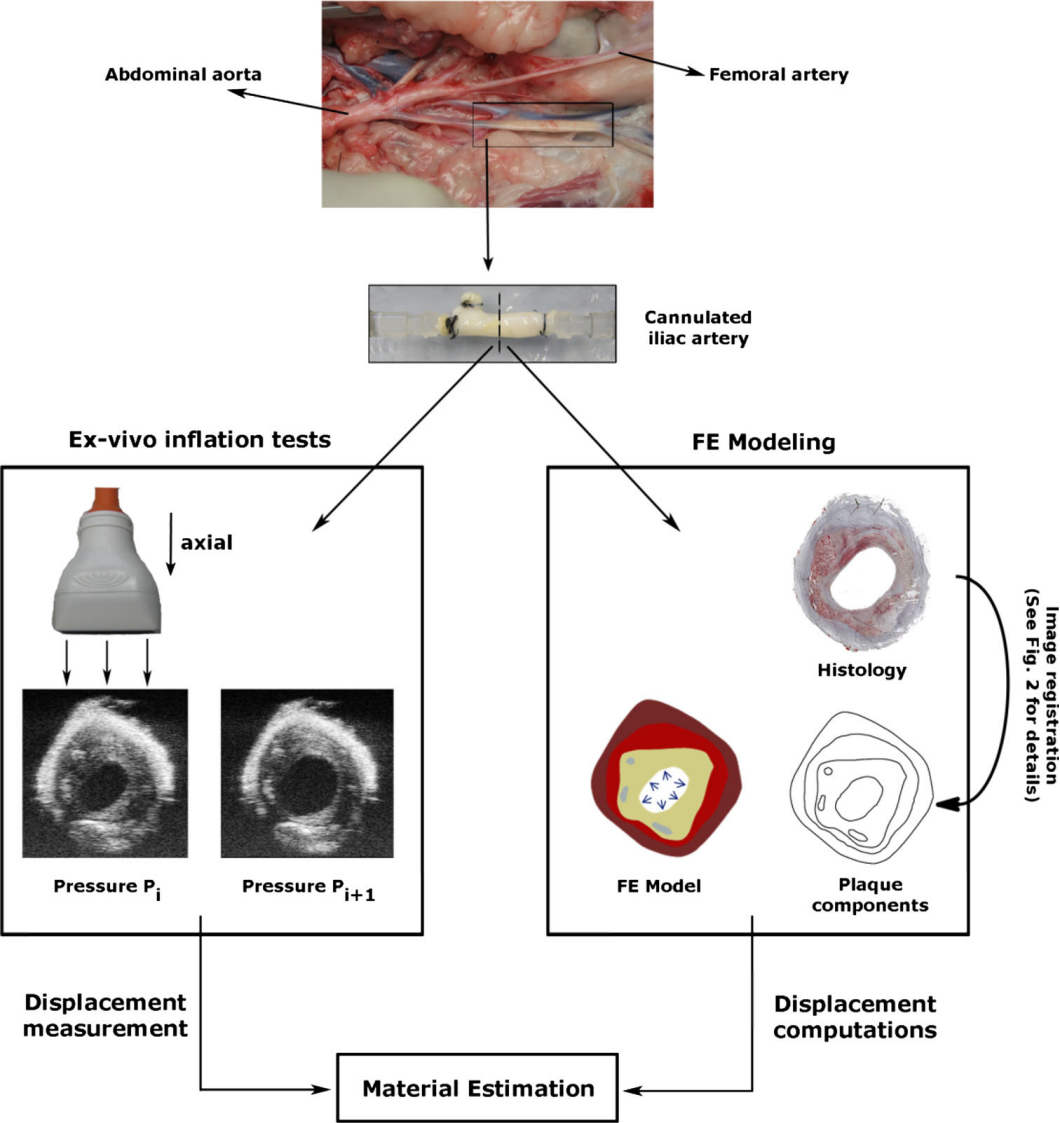
Methodology of the study consists of three main parts: *ex vivo* inflation tests, FE modeling, and estimation of material properties.

The methodology of the study consists of three main parts: *ex vivo* inflation tests, FE modeling, and estimation of material properties of plaque components (Fig. 1). The individual steps are explained below in details.

### *Ex* *Vivo* Inflation Tests

Atherosclerotic iliac arteries (*n* = 6) were collected from diabetic pigs that were on a high cholesterol diet. Briefly, streptozotocin-induced diabetic pigs (~45 kg) were fed supplemental (40% of dietary energy) saturated fat/cholesterol, unsaturated fat or starch for 10 weeks. The pigs showed substantial amount of atherosclerotic lesions in the arterial system. Details of this animal model can be found in previous work.^24^

Immediately after sacrificing the animals, iliac arteries were excised and snap-frozen in liquid nitrogen. Arteries were stored at −80 °C until the day of inflation experiments. At the day of experimentation, the arteries were thawed to room temperature, cannulated and the side branches were closed by sutures. Based on the estimated *in vivo* length, the arteries were stretched 20% in longitudinal direction and preconditioned between 80 and 120 mmHg for ten times. In the inflation experiments, intraluminal pressure was increased from 10 to 120 mmHg in a quasi-static nature and measurements were acquired when the pressure and deformation stabilized. The tests were conducted in a custom build vessel perfusion system at room temperature.

Deformation of the atherosclerotic vessel wall during the inflation test was imaged with a Vevo^®^ 2100 ultrasound system (FUJIFILM VisualSonics, Inc., Toronto, Canada) using a high frequency linear array transducer (MS550D with center frequency of 40 MHz). Before the inflation experiments, a 3D reconstruction of the segment was generated by stacking a series of 2D B-mode images using a 3D motor stage. The images were evaluated visually to identify a location with a substantial atherosclerotic plaque, at least 10 mm away from either cannula. The location was marked with a tissue marker pen on the outside of the vessel to find it accurately in a later phase for histology preparation. Transversal cross-sectional B-mode ultrasound images and radiofrequency (RF) data were recorded at this location at each pressure step.

The RF data enabled us to calculate the axial (along the ultrasound lines) and lateral (in the direction perpendicular to ultrasound lines) displacements on the transversal plaque cross-section with high precision. Due to the availability of phase and amplitude information in axial direction, the displacement estimates in this direction are more accurate than in the lateral direction. The displacement estimation is performed in three iterations with a coarse-to-fine 2D cross-correlation method using RF data and is explained in detail elsewhere.^29^ The displacement estimation method was originally developed for clinical ultrasound scanners using center frequencies up to 10 MHz, and applied for high-frequency scanners for the first time. In the first iteration, template and search kernel sizes of 3850 × 715 *µ*m^2^ (axial × lateral) and 7700 × 935 *µ*m^2^ with a kernel overlap of 50% in the axial direction and a kernel overlap of 92% in the lateral direction were used to find a coarse displacement estimate. In the second iteration template and search windows sizes of 60 × 715 and 120 × 935 *µ*m^2^ were used with a window overlap of 75% and 92% in the axial and lateral direction, respectively. In the last iteration local aligning of the data including parabolic interpolation of the cross-correlation peak was performed to obtain accurate subsample displacement estimates. The kernel sizes remained the same in iteration two and three. A median filter of 5 × 5 displacement pixels was applied after each iteration to decrease the amount of outlying displacement values. The final spatial resolution for the displacement estimates was 15 *µ*m in axial direction and 55 *µ*m in the lateral direction.

### Finite Element Models

**Figure 2:**
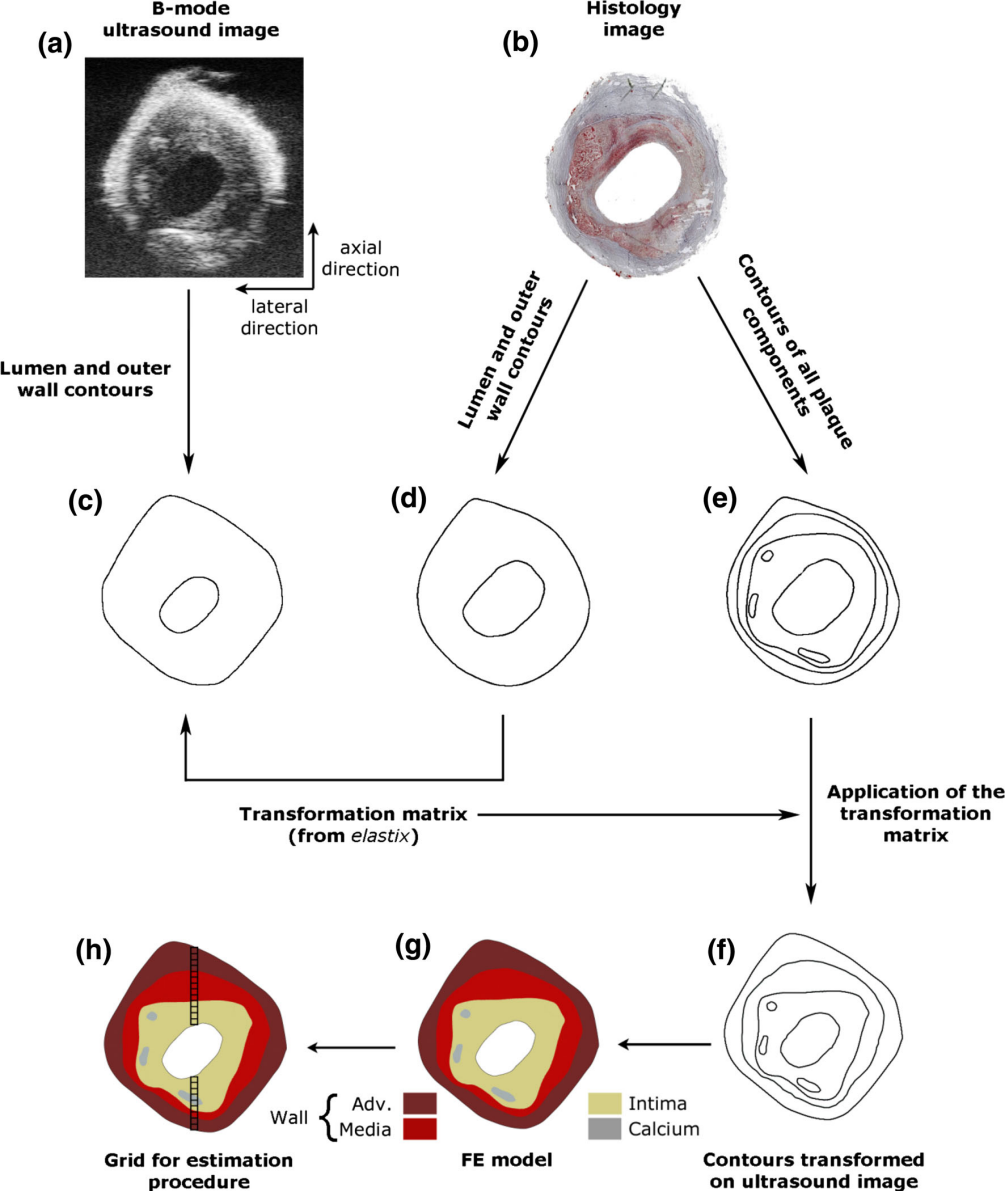
Illustration of how the geometry for FE models was obtained. Plaque component borders drawn on histology images were transformed onto the B-mode ultrasound image by the image registration software *elastix.* Axial (along the ultrasound lines) and lateral (in the direction perpendicular to ultrasound lines) directions are indicated next to the B-mode image.

Plaque morphology is crucial in FE models if mechanical characterization of individual plaque components is aimed for. Ultrasound imaging can provide this essential information to a certain degree, as sufficient contrast cannot be obtained to delineate all plaque components. Although lumen and outer vessel wall contours can be detected clearly and easily, media-intima interface and calcium regions could not be identified precisely. To overcome this problem, we used a hybrid approach in which histology images provide the detailed information of different plaque components (Fig. 2). To obtain the histological images, the arteries were fixed with formaldehyde for histology at 10 mmHg and 20% longitudinal pre-stretch. One 5-*μ*m thick slice from each imaged plaque cross-section was used for Oil red O (ORO) staining, counter stained with hematoxylin. In ORO staining, lipids and fatty acids appear red. The counter-staining hematoxylin stains the nuclei and calcium blue. This staining enabled us to delineate the adventitia, media, intima, calcium, lumen and the outer border of the vessel wall.

The histological images were slightly different in shape and size than the B-mode images due to histology preparation. To ensure that the plaque morphology in the FE models accurately reflects the plaque morphology in the inflation tests, the histology images were registered to B-mode images. Contours of plaque components delineated on histology images were mapped on ultrasound images (Figs. 2c–2f) using the image registration software elastix.^23^ elastix is an open source software, based on the well-known Insight Segmentation Toolkit (ITK) and used for (medical) image registration. First, the lumen contour and the outer vessel wall contour were manually drawn both on the ultrasound image obtained at 10 mmHg (Fig. 2c) and on the histology image (Fig. 2d). Subsequently, the contours on the images were mapped. This resulted in a transformation matrix from histology image to ultrasound image for the entire plaque cross-section. Finally, the contours of the plaque components drawn on the histology image (Fig. 2e) were transformed onto the ultrasound image (Fig. 2f) by using the transformation matrix obtained in the previous step. The transformed histology contours were used to create 2D FE models of the plaques with detailed morphology information (Fig. 2g).

Based on the histology images, FE models (ABAQUS, version 6.11) were created to simulate the plaque deformation during the inflation tests. The nonlinearity in the material behavior of plaque components was modeled with a nonlinear, incompressible Neo-Hookean model for all plaque components. Incompressible Neo-Hookean material model is characterized by the strain energy density function *W* defined as *W* = *C* (*I*_1_ − 3), where *C* is the shear modulus and *I*_1_ is the first invariant of the left Cauchy-Green deformation tensor. Identical shear moduli were assigned to adventitia and media. This material complex is referred to as “wall” in the rest of the paper. Calcium was assumed to be very stiff (*C* = 10^5^ kPa). To prevent rigid body motion in the FE simulations, a very soft and compressible solid buffer layer surrounding the plaque was created and zero-displacement boundary conditions were applied to its outer border. The models were pressurized intraluminally following the protocol of the inflation experiment and solved using previously developed numerical procedures.^2^,^41^ Linear triangular and quadrilateral plane strain elements were used in the FE models. Hybrid elements were preferred to avoid volumetric locking. The total number of elements in each model differed depending on the size and shape of the plaque cross-section and varied between 20 × 10^3^ and 100 × 10^3^. The FE models passed the checks on convergence and satisfaction of applied load and boundary conditions.

### Estimation of Material Properties

A grid search method was employed for the material parameter estimation. The material parameters were altered within a prespecified range with a constant step size and all possible combinations of the parameters were simulated. In the estimation procedure, shear moduli of plaque wall and intima were varied between 1 and 400 kPa in the FE models.

The measured displacements from central region of the plaques in the axial direction were used in the estimation procedure to find the optimum material properties. The axial direction of the ultrasound beam in this region corresponds to the radial direction in polar coordinates with the coordinate system center at the lumen center. Plaques were located in the test setup such that the thickest plaque section was in this region. A grid with 100 *µ*m element size (~6× the axial and ~2× the lateral in-plane resolution of ultrasound data) was generated in this plaque region as illustrated in Fig. 2h. The total number of elements in the grids varied between 30 and 50 depending on the plaque thickness.

In each grid element, both the computed and measured displacements were averaged. To cover the material nonlinearity in the physiological pressure range in arteries of humans (80 to 120 mmHg), the displacements from 10 to 80 mmHg (step 1), from 80 to 100 mmHg (step 2), and from 100 to 120 mmHg (step 3) were used in the analysis. An objective function, *F*, to be minimized in the estimation procedure for each pressure step was defined as

$$F = \sum\nolimits_{j = 1}^{n} {\left( {u_{j}^{\text{comp}} - u_{j}^{\text{meas}} } \right)^{2} ,}$$

similar as was done by Beattie *et al.*^4^ In the objection function, *F*, $u_{j}^{\text{comp}}$ and $u_{j}^{\text{meas}}$ are the average computed and measured displacement in each grid, respectively, “*j*” represents the grid element number and “*n*” the total number of the grid elements. The FE simulation with the lowest value of the objective function, *F*, was considered as the best match to the experimental measurements and the shear moduli used in this FE model as the estimate of the intima and wall material properties. The estimation procedure was run for all 3 pressure steps separately to have shear modulus estimation for each pressure step. The corresponding Young’s modulus values, *E*, of the estimated shear moduli were calculated with the formula, *E* = 6 * *C*, and reported in the remainder of the paper for an easy comparison to the results reported in literature. The goodness of the parameter estimation for each plaque and each pressure step was evaluated by computing the relative difference between the measured and computed displacement:

$$\varDelta u_{\text{rel}} = \left[ {\left( {\frac{\sqrt F }{n}} \right)\left( {u_{\text{mean}}^{\text{meas}} } \right)^{ - 1} } \right] \times 100\% ,$$

where $u_{\text{mean}}^{\text{meas}}$ represents the average absolute displacement for each pressure step.

## Results

**Figure 3:**
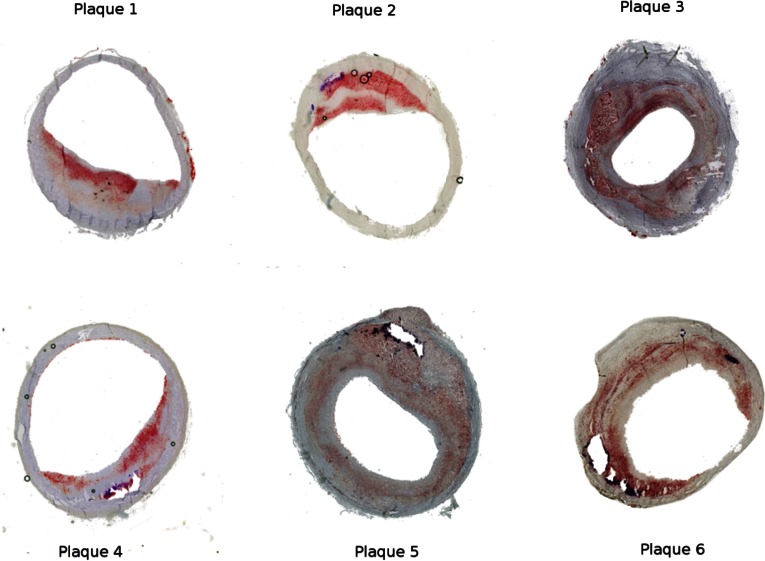
Histology images of the atherosclerotic plaques from porcine iliac arteries. The holes with the blue outline in the images are where calcifications were present before they dissolved during the histology preparation.

Of the six porcine iliac plaques, two plaques were concentric and four were eccentric. Figure 3 shows the histology images of the plaques. The atherosclerotic intima contained extracellular lipids and collagen fibers. No lipid pool or necrotic core was present in any of the plaques. All but plaque 1 contained calcium in varying sizes. The calcifications were localized usually near the intima-media interface.

**Figure 4:**
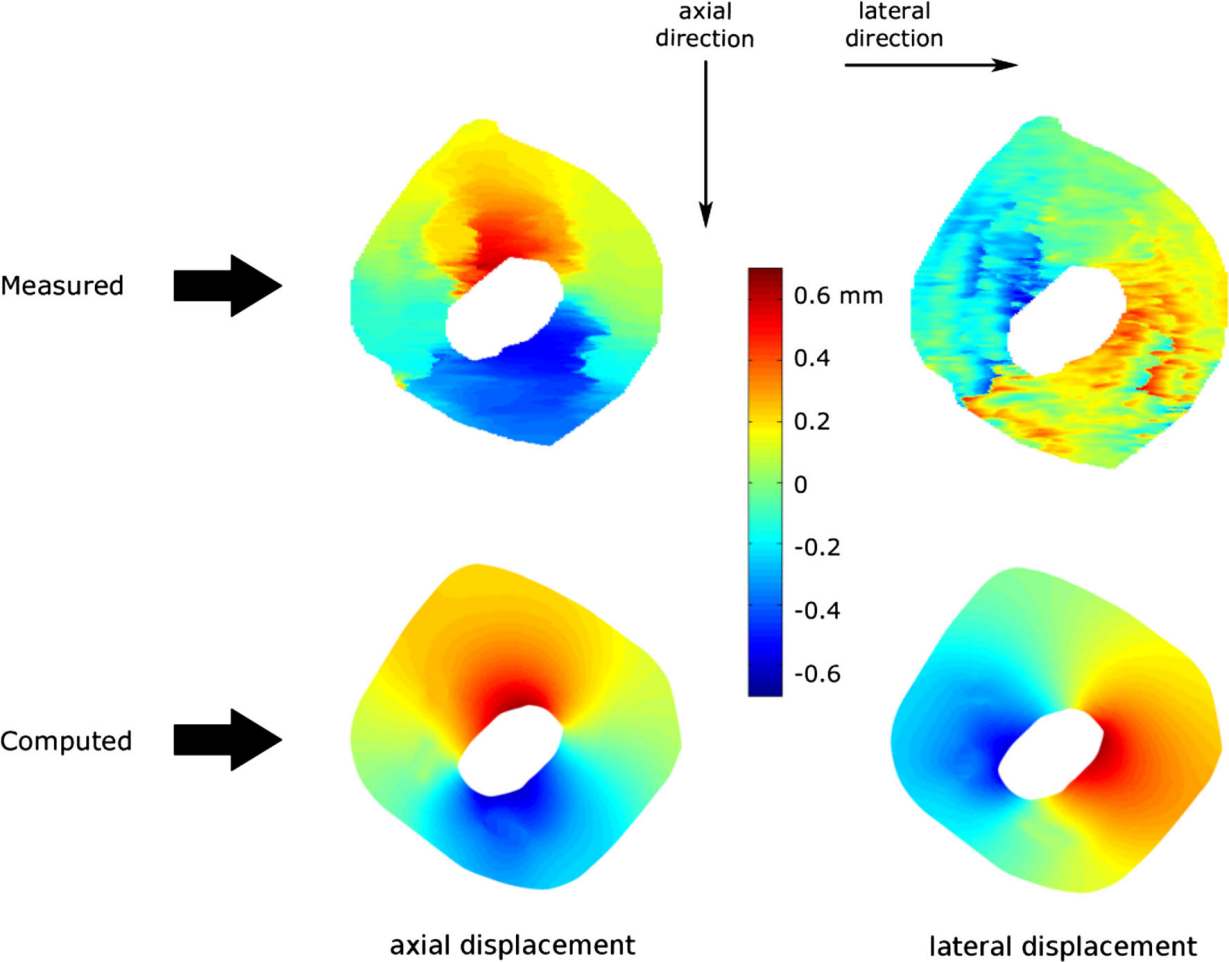
Measured (upper panel) and computed (lower panel) displacement maps in the axial direction (parallel to the ultrasound beam) in the left panel and lateral direction (perpendicular to the ultrasound beam) in the right panel for plaque 3 due to intraluminal pressure increase for pressure step 1 (from 10 to 80 mmHg).

Displacement measurements were successfully performed with the cross correlation technique applied to the RF data from high frequency ultrasound imaging. An example (Plaque 3) of the measured displacements from 10 to 80 mmHg in the axial and lateral directions is shown in the upper panel of Fig. 4. Axial displacements showed a smooth profile. As expected, the lateral displacement measurements were less smooth. The corresponding computed displacements (of the simulation with the minimum objective function value) are shown in the lower panel of Fig. 4. The figure demonstrates that the principal displacements within the plaque were outwards in the radial direction, which is the expected deformation profile of a pressurized arterial segment.

**Figure 5:**
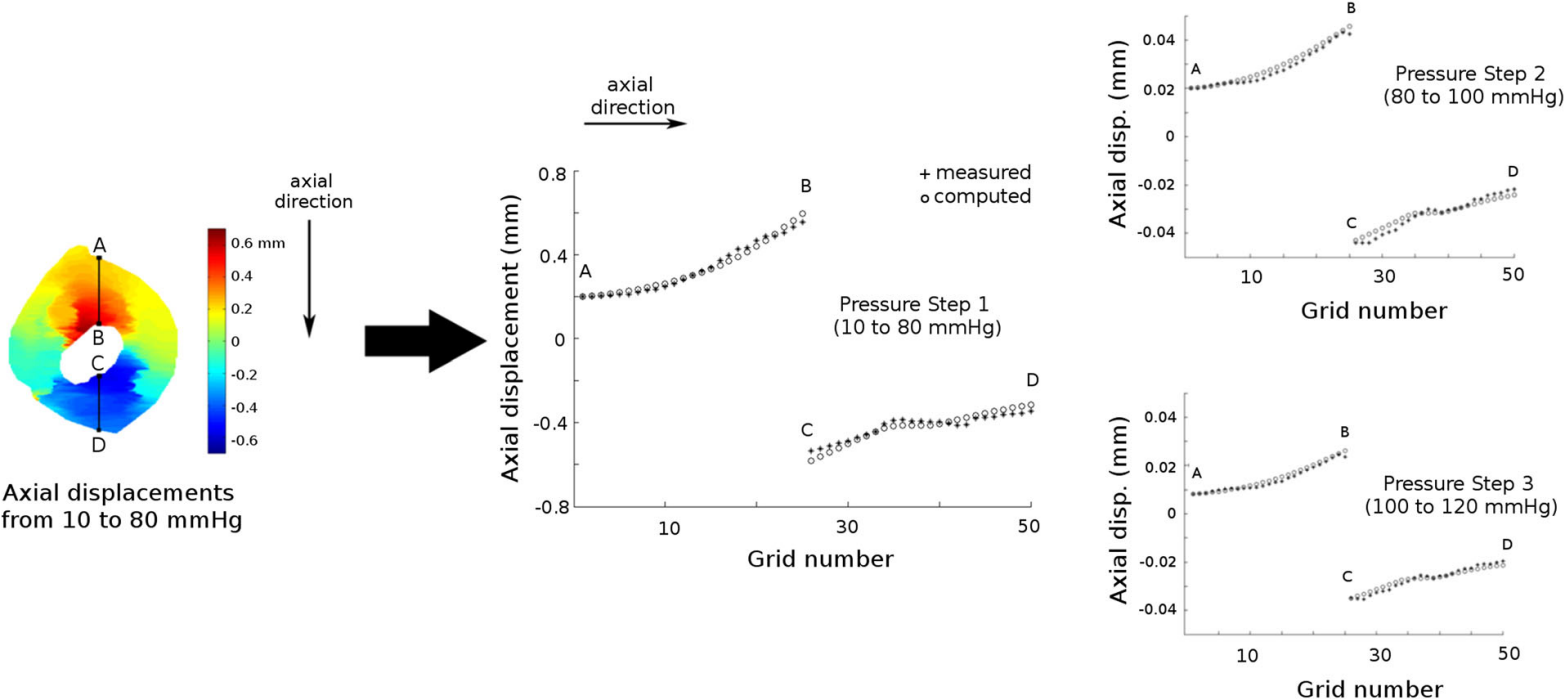
Measured (solid dots) and computed (circles) axial displacements in the midsection of plaque 3 due to intraluminal pressure increase during inflation. Please note the difference in the *y*-axis scaling for the three pressure steps.

Figure 5 represents the measured and computed axial displacements in the grid elements (lines A–B and C–D) for all three pressure steps for plaque 3. For pressure step 1, the displacement of point A was measured as +0.20 mm whereas point B displaced by +0.56 mm. This implies that the tissue between point A and B was compressed by 0.36 mm. Similarly, the tissue between the point C and D was compressed by 0.20 mm. The computed displacements showed a very good agreement with the measured displacements. For this plaque and pressure step 1, the relative difference between the measured and computed displacement, Δ*u*_rel_, was 0.8%. Not only the general deformation pattern, but also local deformations were captured with both the ultrasound measurements and FE simulations. The plateau in the measured displacements between the points C and D suggests that this region showed a relatively small compression. Inspection of the histology and ultrasound images confirmed the presence of calcium in this region (Fig. 2). For pressure steps 2 and 3, the measured displacements were lower than for pressure step 1 as the pressure increase for step 2 and 3 was lower. The tissue region between A and B was compressed by 0.023 mm during pressure step 2 and by 0.019 mm during pressure step 3. The lower compression in pressure step 2 compared to step 3 indicates that the tissue stiffened as the pressure increased. Δ*u*_rel_ for pressure steps 2 and 3 were 0.9 and 0.8%, respectively. For all plaques combined, the average [range] Δ*u*_rel_ for pressure step 1, 2 and 3 were 1.1% [0.5–1.2%], 2.7% [0.9–6.4%], and 2.4% [0.8–6.2%], respectively. Out of 18 cases (three pressure steps for six plaques), 14 had Δ*u*_rel_ lower than 2% whereas the remaining four steps (step 2 and 3 for plaques 1 and 4) had Δ*u*_rel_ greater than 4.5%.

Table 1 displays the estimated Young’s modulus values for atherosclerotic intima and wall for the 6 models together with the mean values. Both plaque components showed increasing stiffness with increasing pressure. The mean Young’s modulus (±standard deviation) for intima was 24 (±17) kPa for the pressure change from 10 to 80 mmHg, 100 (±68) kPa for the pressure change from 80 to 100 mmHg, and 190 (±187) kPa for the pressure increase from 100 to 120 mmHg. On average, arterial wall was stiffer than intima for all pressure steps. For the wall, mean Young’s modulus values increased from 142 (±108) to 877 (±459) kPa, and then to 1015 (±439) kPa for the same sequence of pressure steps. The estimated stiffness for the individual plaques showed a wide variation but consistent behavior. Two exceptions were observed: for plaque 1, the stiffness of the wall slightly decreased when going from pressure step 2 to step 3. For plaque 4, the intima showed very low stiffness values, hitting the lower limit for pressure step 2 and 3. It should be noted that these four cases are the ones that resulted in larger Δu_rel_ values (>4.5%) compared to the other 14 cases (<2%). If these 4 cases are excluded from the analysis, the mean Young’s modulus (±standard deviation) for intima was calculated 135 (±47) kPa for the pressure step 2, and 149 (±39) kPa for the pressure step 3, and for the atherosclerotic arterial wall, 678 (±332) kPa for the pressure step 2, and 879 (±290) kPa for the pressure step 3.

**Table 1:** Estimated Young’s modulus values of the intima and wall of the atherosclerotic plaques (in kPa).

	Intima			Wall		
	Pressure Step 1 (10–80 mmHg)	Pressure Step 2 (80–100 mmHg)	Pressure Step 3 (100–120 mmHg)	Pressure Step 1 (10–80 mmHg)	Pressure Step 2 (80–100 mmHg)	Pressure Step 3 (100–120 mmHg)
Plaque 1	48	60^a^	546^a^	108	924^a^	792^a^
Plaque 2	18	72	90	36	528	696
Plaque 3	30	138	162	48	348	570
Plaque 4	6	1^a^	1^a^	186	1626^a^	1782^a^
Plaque 5	4	186	174	330	1122	1164
Plaque 6	36	144	168	144	714	1086
Mean ± SD	24 ± 17	100 ± 68	190 ± 187	142 ± 108	877 ± 459	1015 ± 439

The cases whose Young’s modulus values are indicated with superscript letter (a) where Δ*u*_rel_ values were significantly higher than the rest (>4.5% vs. <2%)

## Discussion

This study presented a new hybrid experimental–numerical approach to determine local mechanical plaque properties. An RF-correlation technique was applied to high frequency ultrasound data to quantify plaque deformation at a high spatial resolution during inflation. Experiments with intact arteries enabled testing plaques with preserved structural integrity. By registering histology images to ultrasound images unprecedented details on complex plaque morphology were incorporated in the FE models. These models were used in an inverse FE method technique to estimate the material parameters of the arterial wall and intima of atherosclerotic plaques. This novel technique was utilized to characterize 6 porcine iliac atherosclerotic plaques from *ex vivo* inflation tests.

Accurate quantification of the local displacement distribution is essential for application of inverse methods to map the local properties of complex structures like atherosclerotic plaques. To measure the local displacement fields in the atherosclerotic plaques, a previously developed ultrasound RF cross correlation technique was applied to high-frequency ultrasound data for the first time. This technique was previously validated with vessel phantoms.^17^ An axial displacement estimation RMSE (root-mean-squared error) of 0.63 *µ*m was achieved in this validation study at an ultrasonic signal-to-noise level of 20 dB using a linear array transducer with a central frequency of 7.5 MHz. Although not verified, a smaller RMSE is anticipated with the high frequency ultrasound transducer used in the current study. This RF cross correlation technique combined with high frequency ultrasound resulted in displacement maps with an axial resolution of 15 *µ*m, enabling us to image local heterogeneities over spatial scales that are relevant for atherosclerotic plaques. Not only are these high resolution measurements essential for the current application, they will also be essential if we want to study previously reported inhomogeneities within the intima.^7^

A high accuracy in matching the simulated displacement field to the measured data (Δ*u*_rel_ < 2%) was achieved for 14 cases out 18 cases. This indicates that the underlying plaque model captures the experimental data very well for these cases. This observation has two implications. The first implication is that the registration between the histology data and the ultrasound data, based on only the lumen and outer wall contour information, resulted in a good approximation of the underlying plaque geometry in the central region of the plaque. The second implication is that the neo-Hookean models used for the arterial wall and the intima captured the mechanical response in the central region of the plaque to the given intraluminal pressure very well. Employing a more nonlinear material model was possible, however, not preferred in this study as the aim was to develop a framework to estimate local stiffness values. To demonstrate the feasibility of this framework, a single parameter neo-Hookean material model for the diseased intima and the wall was employed. Fitting a multiparameter nonlinear material model to the strain data may result in overparameterization and therefore nonunique solutions.^6^,^42^,^46^ To evaluate nonlinear stiffening of the tissue, an incremental stiffness assessment was done by fitting the neo-Hookean model to strain data from three incremental pressure steps.

On average, both the intima and the wall exhibited the expected nonlinear behavior:^1^ the stiffness increased with increasing pressure. Furthermore, the average stiffness values for the wall were higher than the stiffness value for the intima component. This indicates that the arterial wall was the major load bearing structure in these atherosclerotic iliac plaques. The average wall stiffness values are similar to healthy porcine aorta wall properties obtained from inflation tests.^22^,^30^ The average intima stiffness values are in the lower range of human data in literature,^1^ and similar to the compressive stiffness values reported for aortic plaques^27^ and tensile stiffness for human carotid intima tissue.^10^ The histology examination of the tested porcine plaques revealed that these plaques show pathological features of early stage human plaques, hence low stiffness results are not surprising. The average data however cannot conceal the considerable variation in the intima and wall properties. Even in plaques, harvested from the same vascular territory from animals of the same age that were exposed to the same diet, stiffness values span a wide range. This implies that for accurate modeling of the stress distribution in atherosclerotic plaques, plaque specific properties should be used.

The new hybrid experimental–numerical approach has the potential to be extended to *in vivo* use. To translate the technique to carotid plaque assessment, high resolution strain/displacement imaging and plaque composition data are required. Resolution of about 0.2 × 0.2 mm^2^, which is not that far from the resolution used in this study, can be obtained if a clinical linear-array transducer with 9 MHz center frequency is used to measure displacement field in carotid plaques. Obtaining high resolution plaque morphology data to replace histology is more challenging, but magnetic resonance imaging (MRI) can provide this information. In a recent study we demonstrated that a comparable approach using ultrasound based strain fields and plaque composition from MRI is fairly robust and insensitive to small segmentation errors.^33^ To determine coronary plaque properties, invasive imaging techniques have to be used as coronary arteries are too small and far from body surface. Intravascular ultrasound virtual histology (IVUS-VH) can be employed to determine the morphology and intravascular ultrasound palpography (IVUS Palpography) to measure the deformation. Combination of the two imaging modalities was utilized before^40^ and can provide the required data set for application of this framework in coronary arteries. Engagement of optical coherence tomography (OCT) can offer further improvement in imaging the luminal side of plaques, which would be very beneficial especially for characterization of thin cap fibro-atheromas.^13^

Some limitations of the method presented in this study should be noted. First, only axial displacements were utilized in the minimization procedure as lateral displacement measurements by ultrasound were shown before to be much noisier than axial displacements.^16^,^29^,^31^ To obtain accurate lateral displacement from ultrasound recordings more advanced displacement estimation techniques such as spatial compounding technique are required,^17^,^25^ which have not been implemented yet for high frequency ultrasound systems such as the one used in our study. We used displacement measurements only from the mid-section of the plaque cross-sections since the most accurate ultrasound displacement measurements are collected in the central region of the ultrasound beam. To test how the stiffness results from the optimization procedure can be affected by the region size, we conducted a pilot study on plaque #3. Using axial displacements not only from the mid-section but the entire plaque cross-section in the optimization did not change stiffness results, only the relative error increased from 0.8 to 4.8% as noisier data from the side regions were included. Same stiffness results when full plaque cross-section incorporated can be explained by the relatively simple morphology of the plaques tested. The qualitative histological investigation showed that the tested porcine iliac plaques were fairly homogeneous. Although this observation corroborates modeling the atherosclerotic intima homogenously for the tested plaques, *in vivo* application of the proposed method to advanced human plaques would require 2D displacement field of the entire plaque cross-section as they are morphologically and possibly also mechanically more inhomogeneous. In the future we plan to study the added value of full 2D displacement measurements to estimating material properties of plaque components and if it is required to model the inhomogeneity of advanced atherosclerotic plaques. Secondly, the software elastix, which was used to register histology images to ultrasound images, employs a purely image based algorithm and does not incorporate any structural, physical information to compute the transformation matrix for the registration. This might lead to some local errors in the registration. However, in our study successful registration for 14 out of 18 plaques was confirmed by the low relative difference between the measured and computed displacement fields in the central region. A representative example of a case with low relative difference can be seen in Fig. 5. Thirdly, possible anisotropic material behavior of intima and wall components was neglected in the study. Yet the isotropic Neo-Hookean model, used in FE simulations, resulted in low relative differences between the measured and computed displacement values. More advanced material models can demonstrate the material behavior of atherosclerotic plaque components better and can be employed in the FE models if needed. Similarly, the method presented in this paper allows including more components in the FE models if desired. For instance, although media and adventitia layers are structurally and mechanically two distinct layers, they are fused into one effective arterial wall component as the primary focus of the study was the demonstration of a new method for mechanical characterization of atherosclerotic plaque components, especially atherosclerotic intima. Yet, the proposed technique allows including them separately in the FE models with the cost of more intensive computations. Plane strain assumption is used in the study. The vessel segments were prestretched longitudinally before inflation and kept at constant length during inflation. No appreciable axial displacements were observed visually during the experiments and qualitative assessment of the B-mode images at different pressures did not show any change in the overall appearance of the imaged cross-section. Good correlation between the RF data collected at different pressure steps also confirms this. Therefore, plane strain assumption seems to be justified for this study. Another limitation of the study is that the residual stresses are neglected. Some interesting approaches were proposed earlier on how to investigate residual strains/stresses in atherosclerotic plaques,^34^,^35^ yet the advancement on this issue is very limited as the atherosclerotic plaque structure is very complex, heterogeneous and nonuniform. Therefore, residual strains/stresses were not incorporated in the current study. The test were performed at room temperature as Schaar *et al*. demonstrated that the difference between the strain data collected from *in vitro* inflation experiments of atherosclerotic coronary arteries at room temperature and body temperature were not statistically different.^38^ Inflation procedure leaves the arterial segment intact, which is one of the main advantages of this method. A disadvantage is that, if the plaque is relatively small, the intima will not have a major impact on the displacement field, and as a consequence, the outcome of the minimization procedure will be relatively insensitive to the stiffness of the intima.

In conclusion, we developed a new hybrid experimental–numerical technique to characterize local mechanical properties of atherosclerotic plaques. The combination of geometrical data from histology and high resolution displacement data from ultrasound was used to quantify the properties of intima and the wall atherosclerotic iliac arteries from six pigs. Both components exhibited nonlinear behavior and on average, the wall was stiffer than the intima. A large variation in the properties of the intima was observed, warranting further research into the relationship between stiffness and compositions of the intima.
